# Different Effects and Mechanisms of Selenium Compounds in Improving Pathology in Alzheimer’s Disease

**DOI:** 10.3390/antiox12030702

**Published:** 2023-03-12

**Authors:** Zhong-Hao Zhang, Jia-Ying Peng, Yu-Bin Chen, Chao Wang, Chen Chen, Guo-Li Song

**Affiliations:** 1 Shenzhen Key Laboratory of Marine Bioresources and Ecology, College of Life Sciences and Oceanography, Shenzhen University, Shenzhen 518060, China; 2 Shenzhen Bay Laboratory, Shenzhen 518118, China; 3 Chemical Analysis & Physical Testing Institute, Shenzhen Center for Disease Control and Prevention, Shenzhen 518055, China; 4 Shenzhen-Hong Kong Institute of Brain Science—Shenzhen Fundamental Research Institutions, Shenzhen 518055, China

**Keywords:** Alzheimer’s disease, Se-methylselenocysteine, selenomethionine, sodium selenate, synaptic deficits, metabolism

## Abstract

Owing to the strong antioxidant capacity of selenium (Se) in vivo, a variety of Se compounds have been shown to have great potential for improving the main pathologies and cognitive impairment in Alzheimer’s disease (AD) models. However, the differences in the anti-AD effects and mechanisms of different Se compounds are still unclear. Theoretically, the absorption and metabolism of different forms of Se in the body vary, which directly determines the diversification of downstream regulatory pathways. In this study, low doses of Se-methylselenocysteine (SMC), selenomethionine (SeM), or sodium selenate (SeNa) were administered to triple transgenic AD (3× Tg-AD) mice for short time periods. AD pathology, activities of selenoenzymes, and metabolic profiles in the brain were studied to explore the similarities and differences in the anti-AD effects and mechanisms of the three Se compounds. We found that all of these Se compounds significantly increased Se levels and antioxidant capacity, regulated amino acid metabolism, and ameliorated synaptic deficits, thus improving the cognitive capacity of AD mice. Importantly, SMC preferentially increased the expression and activity of thioredoxin reductase and reduced tau phosphorylation by inhibiting glycogen synthase kinase-3 beta (GSK-3β) activity. Glutathione peroxidase 1 (GPx1), the selenoenzyme most affected by SeM, decreased amyloid beta production and improved mitochondrial function. SeNa improved methionine sulfoxide reductase B1 (MsrB1) expression, reflected in AD pathology as promoting the expression of synaptic proteins and restoring synaptic deficits. Herein, we reveal the differences and mechanisms by which different Se compounds improve multiple pathologies of AD and provide novel insights into the targeted administration of Se-containing drugs in the treatment of AD.

## 1. Introduction

Alzheimer’s disease (AD), the “second killer” in the diseases of the elderly, is a progressive neurodegenerative disease characterized by synaptic failure and cognitive deficit attributable to amyloid beta (Aβ) oligomers and hyperphosphorylated tau in the brain [1]. Selenium (Se), an essential trace element, gradually decreases with age in the brain and plays an important role in many physiological functions [2], especially in regulating redox homeostasis [3,4]. Moreover, Se deficiency has been reported to occur in patients with neurodegenerative diseases, such as AD and Parkinson’s disease, resulting in the inability of neuronal cells to scavenge excess reactive oxygen species (ROS) effectively, thus affecting the structure and function of protein and DNA molecules, accompanied by multiple cellular homeostatic imbalances [5,6,7].

In nature, Se exists mainly in inorganic and organic forms, and Se supplementation has great potential for AD treatment [8]. Inorganic Se (sodium selenate (SeNa) and sodium selenite (SeNi)) has been reported to play a role in improving cognitive impairment in AD mouse models [9,10,11]. Selenomethionine (SeM) is a stable organic form of Se in the human body [12]. Our previous studies have shown that SeM can significantly improve the learning and memory function of 3× Tg-AD mice and delay the pathological processes related to AD [13]. Some other organic Se molecules, such as Se-methylselenocysteine (SMC) and Ebselen, also exhibit favorable anti-AD effects [14,15].

Indeed, various Se compounds have shown similar therapeutic effects in AD, but the potential mechanisms of action appear to be inconsistent. SeNa was initially found to affect tau hyperphosphorylation by regulating the activity of protein phosphatase 2 (PP2A) [11]. The activation of autophagic flux mediated by SeM was found to promote the clearance of Aβ and tau [16,17], and SMC has been shown to have a positive effect on synaptic abnormalities in AD [18]. Although multiple pathways have been implicated in the amelioration of AD pathologies by Se, the differences and similarities in molecular mechanisms among Se compounds remain to be elucidated. Selenoproteins require Se for their function. Thus, changes in selenoproteins also effectively reflect the activity of Se compounds. In this study, we explored the effects of three Se compounds (SMC, SeM, and SeNa) on selenoprotein expression and brain metabolism in AD mice and studied the mechanism of their participation in AD pathology. We aimed to provide novel insights on the influence of Se on AD, which could lay the foundation for future effective therapeutic strategies.

## 2. Materials and Methods

### 2.1. Animals and Treatments

The 3× Tg-AD mice (B6; 129-Psen1tm1MpmTg (APPSwe, tauP301L) 1 Lfa/J), as described previously [19], were purchased from The Jackson Laboratory (Bar Harbor, ME). They were housed in cages with free access to food and water at 22 ± 2 °C under a 12 h alternating light/dark cycle. In this study, 6-month-old AD mice were treated with SMC (2.77 μg/mL), SeM (2.98 μg/mL), or SeNa (2.87 μg/mL) in drinking water for 4 weeks, and the control mice received normal drinking water and diet. (The average daily water intake of each mouse in our feeding environment was 5 mL). All experiments were performed in compliance with the guidelines approved by the Animal Ethics and Welfare Committee of Shenzhen University (permit number: AEWC-20140615-002).

### 2.2. Morris Water Maze

Morris water maze testing was conducted as previously described [17] after 4 weeks of treatment. Each mouse was trained to locate the hidden platform on four trial days. The mice were allowed to search for the platform for ≤60 s and rest for 30 s on the platform. The time the mice spent finding the platform was recorded as escape latency. At 24 and 72 h after the fourth day of the spatial learning task, the platform was removed. The mice were then subjected to a 60 s probe trial, and the time they spent in each quadrant and the number of times they crossed the original platform location were recorded. All trials were recorded using a computerized tracking system (Smart V3.0, RWD, Shenzhen, China) for subsequent analysis.

### 2.3. Y-Maze Test

The Y-maze test platform was composed of three rectangular arms (numbered 1, 2, and 3) at an angle of 120°. A camera above the box recorded the movement of the mice, and the software automatically recorded the number of times the mice entered different arms. In this experiment, the mice were placed at the end of arm No. 1 to start the experiment, and the sequence of entering the arms was recorded within 3 min.

### 2.4. Open-Field Test

The open-field test was performed in a quiet environment. The mice were placed in the center of the bottom of the box, and they were filmed and timed. During the experiment, the mice were allowed to explore freely in the open field for 3 min, and the number of times spent standing vertically on their hind limbs, the number of grids crossed, and the distance travelled were recorded.

### 2.5. Elevated plus Maze

The mice were placed in the maze from the center lattice facing the closed arm, and their activities were recorded for 3 min. Observation indicators included the number of open-arm entries (two front paws must enter the arm), residence time in the open arm, the number of closed-arm entries, and residence time in the closed arm. The proportions of open-arm stay times and entry times were calculated.

### 2.6. Western Blot Analysis

Each brain was dissected into hippocampal and cortical samples and homogenized in nine volumes of RIPA buffer (Beyotime Biotechnology, Nanjing, China) with protease and phosphatase inhibitors. The protein concentration of each sample was determined using the BCA Protein Assay (Thermo Fisher Scientific, Waltham, MA, USA). Proteins (20 μg) were loaded into each lane of a 10–15% sodium dodecyl sulfate-polyacrylamide gel. After electrophoresis, the proteins were transferred onto 0.45 μm polyvinylidene difluoride membranes (Millipore) at 100 mA for 1.5 h. Then, the membranes were blocked with 5% fat-free milk in Tris-buffered saline for 1 h at 37 °C, followed by incubation with primary antibodies (Table 1) overnight at 4 °C and then horseradish peroxidase-conjugated secondary antibodies (anti-mouse and anti-rabbit, Cell Signaling Technology, Danvers, MA, USA) for 1 h at 37 °C. The immunoreactive bands were visualized with an enhanced chemiluminescence (ECL) kit (FD NeuroTechnologies, Irvine, CA, USA) and scanned for densitometric analysis using an imaging system (Image Station 4000 M; Kodak) and Fiji software (National Institutes of Health, Bethesda, MD, USA). α-Tubulin, β-actin, and GAPDH were used as the loading controls.

### 2.7. Se Levels and ELISA

The levels of Se in the cortexes of the mouse brains were measured using inductively coupled plasma mass spectrometry (ICP-MS) (Agilent 8900 ICP-QQQ). Standard Se solution (GBW(E) 080215, 100 pg/mL) was obtained from the National Standard Material Research Center (Beijing, China). Four samples were collected randomly from each group. The activities of GPx and TrxR in brain homogenates were measured using a GPx assay kit [20] and TrxR assay kit [21], respectively.

### 2.8. LC-MS Metabolomics

Cerebral cortex samples were placed in 2 mL Eppendorf tubes. Twenty microliters of internal standard (Lmuri 2-chlorophenylalanine, 0.3 mg/mL; 17:0 Lyso PC, 0.01 mg/mL, all prepared in methanol) and 400 μL of methanol-water (4:1 *v*/*v*) were added. The mixture was precooled for 2 min in a freezer at −20 °C, two steel balls were added to the tube, and the mixture was treated in a grinder (60 Hz) for 3 min. The mixture was then subjected to ultrasonic extraction in an ice water bath for 15 min and then kept at −20 °C for 30 min before centrifugation for 15 min (13,000 rpm, 4 °C). Three hundred microliters of supernatant were transferred to a liquid chromatography-mass spectrometry (LC-MS) injection vial and evaporated. The sample was vortexed for 0.5 min with 200 μL methanol-water (1:4 *v*/*v*), followed by ultrasound treatment for 3 min. The mixture was kept at −20 °C for 2 h and then centrifuged for 15 min (13,000 rpm, 4 °C). One hundred and fifty microliters of supernatant was filtered through a 0.22 μm filter, transferred to an LC injection vial, and stored at −80 °C until LC-MS analysis.

In this experiment, the results of the quality control (QC) samples were cross-verified seven times. When the unsupervised principal component analysis (PCA) diagram and aggregation degree of the QC samples is high, the experiment has high stability and repeatability. We used UNIFI 1.8.1 to collect the original data. Before pattern recognition, the original data were processed using the metabolomics software Progenesis QI v2.3 (Nonlinear Dynamics, Newcastle, UK) for baseline filtering, peak recognition, integration, retention-time correction, peak alignment, and normalization. The main parameters were as follows: precursor tolerance, 5 ppm; product tolerance, 10 ppm; and production threshold, 5%. The identification of compounds was based on accurate mass number, secondary fragments, and isotope distribution. Qualitative analysis was performed using The Human Metabolome Database (HMDB), LIPID MAPS (v2.3), and METLIN databases. In this experiment, *t*-tests (Student’s *t*-tests) and multiple variation analysis (fold-change analysis) were used to compare the differential metabolites between two groups and describe the concentration or dispersion trend of the samples. Multi-dimensional/single-dimensional analysis was used to screen the differential metabolites between groups. The screening criteria were the variable importance in projection (VIP) value of the first principal component of the orthogonal partial least squares discriminant analysis (OPLS-DA) model (>1) and the *p*-value of the Magi *t*-test (<0.05). The Kyoto Encyclopedia of Genes and Genomes (KEGG) database was used for pathway enrichment analysis. The KEGG ID of differential metabolites was used to analyze pathway enrichment, and the metabolic pathway enrichment results were obtained.

### 2.9. Statistical Analysis

All pairwise comparison datasets were analyzed using Student’s *t*-tests, and multiple groups were compared using one-way or two-way ANOVA followed by Dunnett’s multiple comparison tests. Data are expressed as the means ± SEMs and were analyzed using GraphPad Prism 9 software.

## 3. Results

### 3.1. Cognitive Function

**Figure 1:**
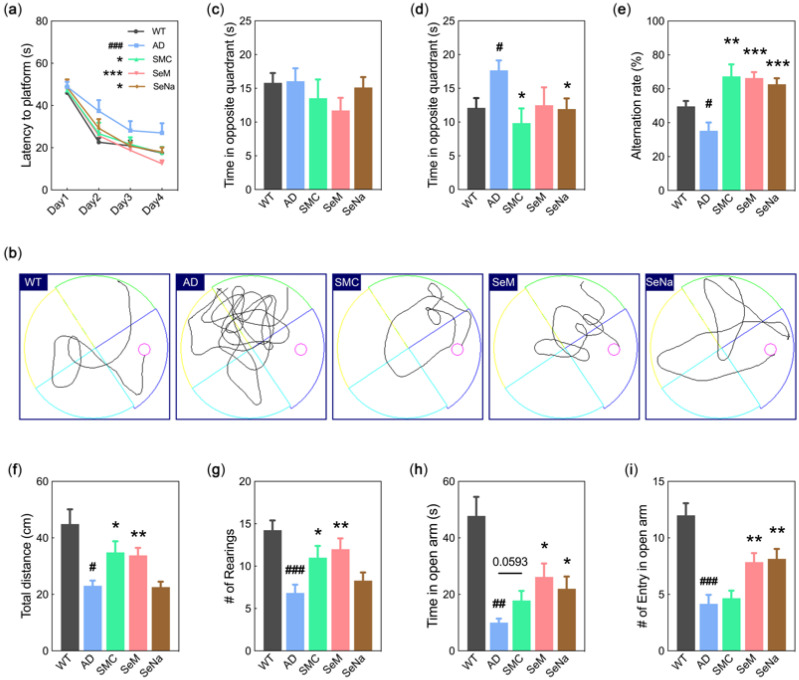
Behavioral tests in the WT, 3× Tg-AD, SMC, SeM, and SeNa mouse groups. (**a**–**d**) The learning and memory abilities of mice were tested using the Morris water maze. (**a**,**b**) The escape latency (**a**) and swimming trajectory (**b**) were recorded during the 4-day training. (**c**,**d**) The probe trial was performed 24 and 72 h after the last trial of a hidden platform task. Time in the opposite quadrant after 24 h (**c**) and 72 h (**d**) was recorded. (**e**) The spatial memory of mice was determined by analysis of the alternation rate in the Y-maze. (**f**,**g**) Total distance (**f**) and number of rearings (**g**) in the open-field test were used to detect the autonomous behavior ability of mice. (**h**,**i**) Mouse anxiety was assessed by the elevated plus maze. Time in the open arm (**h**) and number of entries in the open arm (**i**) were recorded. All data are presented as the means ± SEMs (*n* = 7–10). * *p* < 0.05, ** *p* < 0.01, *** *p* < 0.001 vs. AD mice; ^#^ *p* < 0.05, ^##^ *p* < 0.01, ^###^ *p* < 0.001 vs. WT mice, as determined by one-way or two-way ANOVA followed by Dunnett’s multiple comparison test.

Behavioral analysis was performed to evaluate the therapeutic effects of SMC, SeM, and SeNa in a triple transgenic mouse model of AD (3× Tg-AD mice). Spatial learning ability was evaluated using the time required for the mice to find the hidden platform (escape latency) in the Morris water maze. As shown in Figure 1a, the escape latency of AD mice was significantly longer than that of wildtype (WT) mice, and the performance of AD mice treated with SMC, SeM, and SeNa was significantly better than that of untreated AD mice during four days of continuous training (Figure 1a,b). After a 4-day spatial learning test, a probe trial was used to evaluate the short- and long-term memory (24 and 72 h, respectively) of the mice. The time spent in the opposite quadrant by the AD mice increased significantly in the 72 h memory test (Figure 1d), and this probe time was significantly reduced in the SMC and SeNa groups. There was no significant difference between the groups in the 24 h memory test (Figure 1c,d). The Y-maze was used to test the spatial working memory of mice. Compared to WT mice, AD mice showed a lower spontaneous alternation rate in the confined space of the Y-maze, which was significantly increased in the AD mice treated with SMC, SeM, and SeNa (Figure 1e).

The autonomous behavioral ability and anxiety of mice were further detected by the open-field and elevated plus maze tests. The total distance and rearing number in the open-field test were significantly lower in the AD mice than in the WT mice (Figure 1f,g). SMC and SeM administration significantly increased these parameters in the AD mice (Figure 1f,g). In the elevated plus maze test, the AD mice spent significantly less time in the open arm and made fewer entries into the open arm than the WT mice did, and both SeM and SeNa significantly improved these deficits in the AD mice (Figure 1h,i). The time spent in the open arm also increased with the SMC-treated AD mice, but the changes were not significantly different (Figure 1h). Overall, these data indicate that all three Se compounds can effectively improve the learning and memory ability of 3× Tg-AD mice, but SMC and SeM showed greater efficacy in promoting autonomous behavior, and SeM and SeNa showed better anti-anxiety performance.

### 3.2. AD Pathology

**Figure 2:**
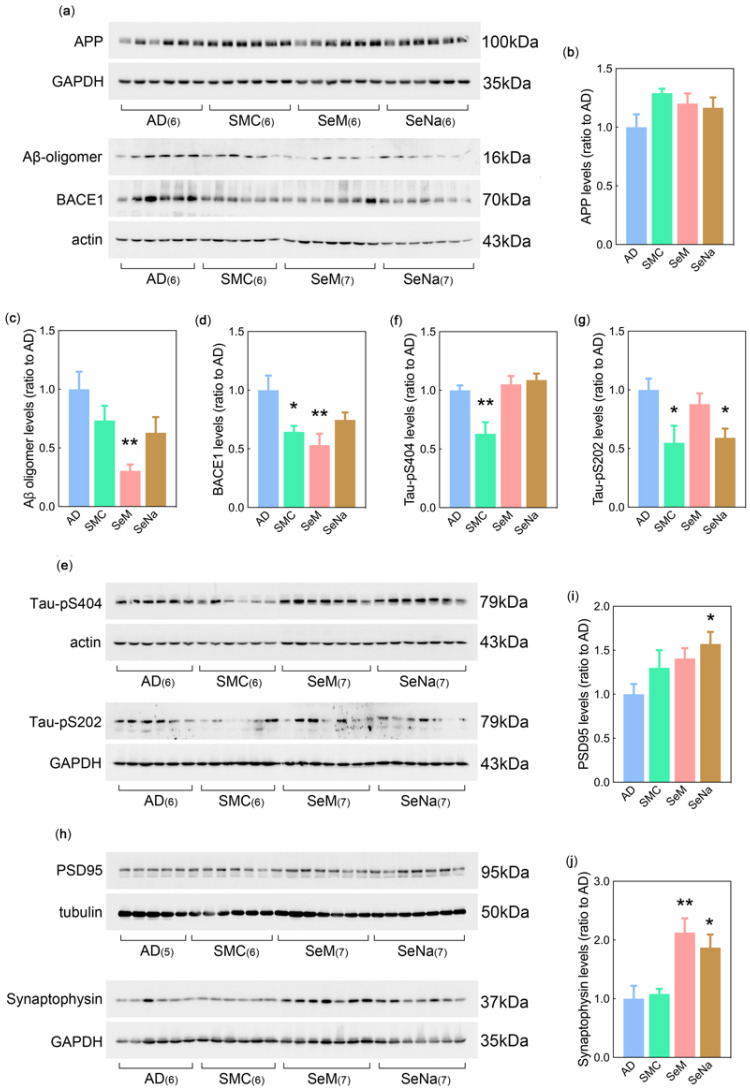
Administration of SMC, SeM, and SeNa improved Aβ, tau, and synaptic pathology in the cortexes of 3× Tg-AD mouse brains. (**a**) Levels of APP, Aβ oligomer (16 kDa), and BACE1 were analyzed by Western blotting. (**b**–**d**) Quantification of protein levels in (**a**). (**e**) Levels of hyperphosphorylated tau (Ser202 and Ser404) were analyzed by Western blotting. (**f**,**g**) Quantification of protein levels in (**e**). (**h**) Levels of PSD95 and synaptophysin were analyzed by Western blotting. (**i**,**j**) Quantification of protein levels in (**h**). α-Tubulin, β-actin, or glyceraldehyde 3-phosphate dehydrogenase (GAPDH) were used as loading controls. All data are presented as the means ± SEMs (*n* = 5–7). * *p* < 0.05, ** *p* < 0.01 vs. AD mice, as determined by one-way ANOVA followed by Dunnett’s multiple comparison test.

To further explore the effects of SMC, SeM, and SeNa on AD pathology, the expression levels of proteins involved in Aβ, tau, and synaptic pathology were detected in the brains of 3× Tg-AD mice. Western blot analysis showed no significant differences in the brain levels of amyloid precursor protein (APP) among all groups of AD mice (Figure 2a,b). However, SeM treatment significantly lowered the levels of Aβ oligomer (16 kDa) in the brains of AD mice, and a decreasing tendency was observed in mice treated with SMC and SeNa (Figure 2a,c). In addition, the expression level of β secretase (BACE1), a key secretase that regulates the cleavage of APP to produce Aβ, was significantly reduced in the brains of AD mice treated with SMC or SeM (Figure 2a,d). Analysis of tau pathology showed that SMC significantly decreased the phosphorylation levels of tau at both Ser404 (tau-pS404) and Ser202 (tau-pS202) (Figure 2e–g). Significantly decreased levels of tau-pS202 were also found in SeNa-treated AD mice (Figure 2e,g). Therefore, SeM and SMC performed better in improving Aβ and tau pathologies, respectively. In terms of postsynaptic density protein 95 (PSD95) and synaptophysin levels, Western blot analysis showed that SeNa significantly promoted the expression of these two proteins in the brains of AD mice (Figure 2h–j), implying that SeNa may have a more significant role in ameliorating synaptic deficits in AD, although synaptophysin levels were also increased in the brains of SeM-treated AD mice (Figure 2h,j).

### 3.3. Se Levels, Selenoenzyme Activity, and Selenoprotein Expression

**Figure 3:**
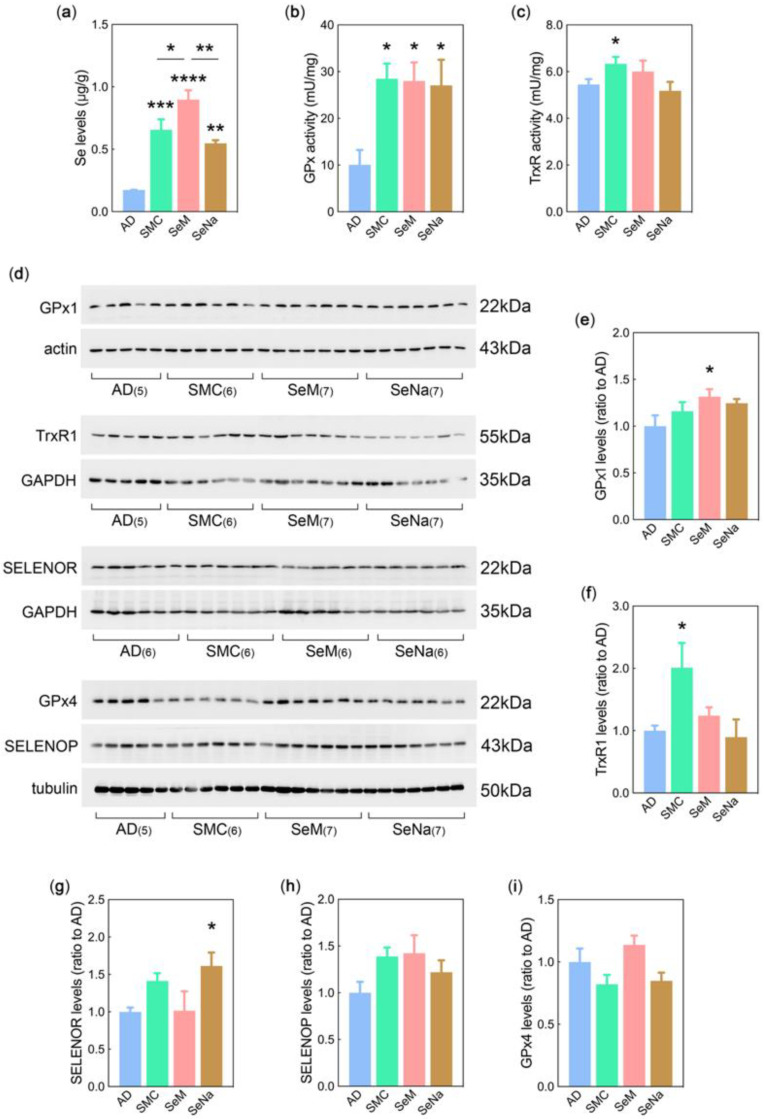
Increases in Se levels, selenoenzyme activity, and selenoprotein expression in the cortexes of 3× Tg-AD mouse brains upon SMC, SeM, and SeNa administration. (**a**) Levels of Se were measured by ICP-MS (*n* = 3–4). (**b**,**c**) Activities of GPx (**b**) and TrxR (**c**) were detected using specific assay kits (*n* = 5–7). (**d**) Levels of GPx1, TrxR1, SELENOR, SELENOP, and GPx4 proteins were analyzed by Western blotting (*n* = 5–7). (**e**–**i**) Quantification of protein levels in (**d**). α-Tubulin, β-actin, or GAPDH were used as loading controls. All data are presented as the means ± SEMs. * *p* < 0.05, ** *p* < 0.01, *** *p* < 0.001, **** *p* < 0.0001 vs. AD mice, as determined by one-way ANOVA followed by Dunnett’s multiple comparison test.

After treatment with SMC, SeM, and SeNa, Se levels in the brains of 3× Tg-AD mice were determined using inductively coupled plasma mass spectrometry (ICP-MS). Compared with control mice, all three Se compounds significantly increased Se levels in the brain; SeM exhibited a more significant ability to increase Se in the AD mice (Figure 3a). Generally, Se performs its biological functions through selenoenzymes or selenoproteins. The results showed that SMC administration significantly increased the activities of glutathione peroxidase (GPx) and thioredoxin reductase (TrxR), vital selenoenzymes that regulate redox status in vivo, in AD mice (Figure 3b,c). In addition, both SeM and SeNa significantly increased GPx activity in the brains of AD mice (Figure 3b).

It has been shown that different selenoproteins exhibit different tissue-specific expression patterns, and our previous analysis regarding the expression and function of 25 selenoproteins in the brain revealed that selenoproteins or selenoenzymes with powerful antioxidant capacities, such as GPx1, GPx4, TrxR1, methionine sulfoxide reductase B1 (MsrB1, previously called SELENOR), and selenoprotein P (SELENOP), were highly expressed in the brain [22]. Therefore, Western blot analysis of these selenoproteins in the brain showed that the level of GPx1 was significantly increased upon SeM administration, while SMC significantly promoted the expression of TrxR1 (Figure 3e,f). In addition, brain SELENOR levels were significantly higher in the SeNa-treated mice than in the AD mice (Figure 3g). However, no significant differences in the levels of SELENOP and GPx4 were observed between the AD control and Se-treated groups (Figure 3h,i). These results demonstrate that SMC, SeM, and SeNa increase Se levels and selenoenzyme activity in the brains of AD mice, but the selenoproteins they affect may vary.

### 3.4. Metabolic Profile in the Brain

There is increasing evidence that Se is involved in various metabolic pathways and that AD is also a metabolic disorder. To further evaluate the effects and mechanisms of the three drugs on AD, we used LC-MS-based untargeted metabolomics to analyze the metabolic profiles of the brains of the 3× Tg-AD mice. PCA was used to evaluate the stability of the system, and the degree of polymerization of the QC samples obtained from the PCA model diagram was high. Using OPLS-DA to maximize the differences between different groups within the model, no fitting phenomenon occurred (Figure S1), indicating that the model of metabolite differences describes the experimental samples well and conforms to the requirement for further analysis.

**Figure 4:**
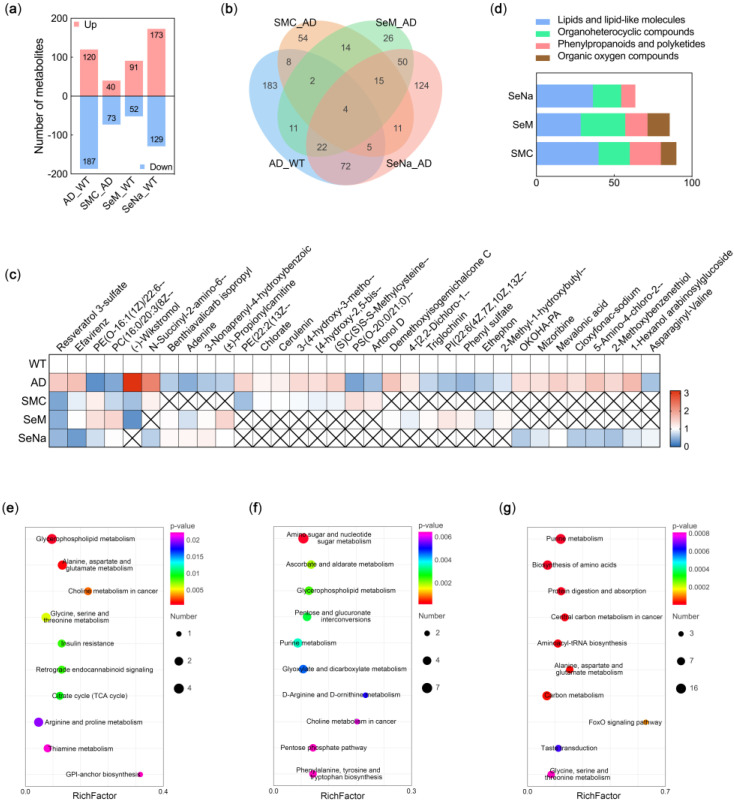
LC-MS untargeted metabolomic analysis revealed altered metabolites and metabolic pathways in the comparisons of AD VS WT, SMC VS AD, SeM VS AD, and SeNa VS AD (*n* = 6 mice). (**a**) Number of upregulated and downregulated metabolites in the four comparisons. (**b**) Venn diagram with the number of differential metabolites in the four comparisons. (**c**) Heatmap of reversed metabolites in the four comparisons. (**d**) Superclass of reversed metabolites with administration of the three Se compounds. (**e**–**g**) KEGG enrichment analysis of differential metabolites in the three comparisons of SMC VS AD, SeM VS AD, and SeNa VS AD.

According to the variable importance in projection (VIP) in the OPLS-DA analysis, a combination of multi-dimensional and one-dimensional analyses was adopted. Three hundred and seven metabolites (120 upregulated and 187 downregulated) in the AD mice were significantly different from those in the WT mice (Figure 4a). As for the comparison groups of SMC VS AD, SeM VS AD, and SeNa VS AD, 113 (40 upregulated and 73 downregulated), 143 (91 upregulated and 52 downregulated), and 302 (173 upregulated and 129 downregulated) differential metabolites, respectively, were identified, most of which were lipids and lipid-like molecules, organoheterocyclic compounds, phenylpropanoids and polyketides, and organic oxygen compounds (Figure 4d). Further analysis of the differential metabolites showed that SMC, SeM, and SeNa reversed the levels of 14, 16, and 17 pathologically differential metabolites, respectively, in AD mice (Figure 4b). As shown in Figure 4c, resveratrol 3-sulfate, efavirenz, PE(O-16:1(1Z)/22:6(4Z,7Z,10Z,13Z,16Z,19Z)), and PC(16:0/20:3(8Z,11Z,14Z)) were the most common reversed metabolites in the three Se-compound treatment groups.

To explore the prevalence and characteristics of the effects of Se compounds on AD mice, we conducted a specific analysis of the association of the differential metabolites with AD (Figure 4c). Among the differential metabolites that were decreased in the AD mice and reversed by SMC, flavonoid metabolites (artonol D) have been shown to be involved in reducing tau hyperphosphorylation [23]. Amino pyrimidine metabolites (2-methyl-1-hydroxybutyl-ThPP), terpenes (3-nonaprenyl-4-hydroxybenzoic acid), and chalcone (demethoxyisogemichalcone C) related to the aggregation and degradation of Aβ are also known to be differential metabolites reversed by SeM [24,25,26]. We continued to analyze the decreased metabolites reversed by SeNa and found published reports that acylcarnitine administration increases choline synthesis and release in the synaptosomes, striata, and hippocampi of rats [27,28]. In addition, adenine and carbamate (benthiavalicarb-isopropyl) have been shown to affect synaptic neurotransmitters [29].

In the Kyoto Encyclopedia of Genes and Genomes (KEGG) enrichment analysis (Figures S2 and S3), the most common metabolic pathways among the top 10 metabolic pathways reversed by the three Se compounds were amino acid-related. Alanine, aspartate, and glutamate metabolism; glycine, serine, and threonine metabolism; arginine and proline metabolism; and phenylalanine, tyrosine, and tryptophan biosynthesis were significantly affected (Figure 4e–g). Interestingly, we also found that SMC, SeM, and SeNa might improve pathology of the AD mouse model through their unique metabolic pathways. Among them, the thiamine metabolism and insulin resistance pathways enriched by differential metabolites of SMC VS AD share a common downstream protein GSK-3β (Figure 4e), which is involved in tau phosphorylation [30,31,32]. In contrast, the differential metabolites of SeM VS AD were enriched in glyoxylate and dicarboxylate metabolism related to mitochondrial function and Aβ aggregation [33,34,35] (Figure 4f). In the pathways enriched by differential metabolites of SeNa VS AD, the Foxo (Figure 4g), AMPK, and mTOR signaling pathways all implicate Foxo6, a protein associated with memory consolidation and synaptic function [36,37,38,39].

### 3.5. Mechanisms of Se Compounds in Improving AD Pathology

**Figure 5:**
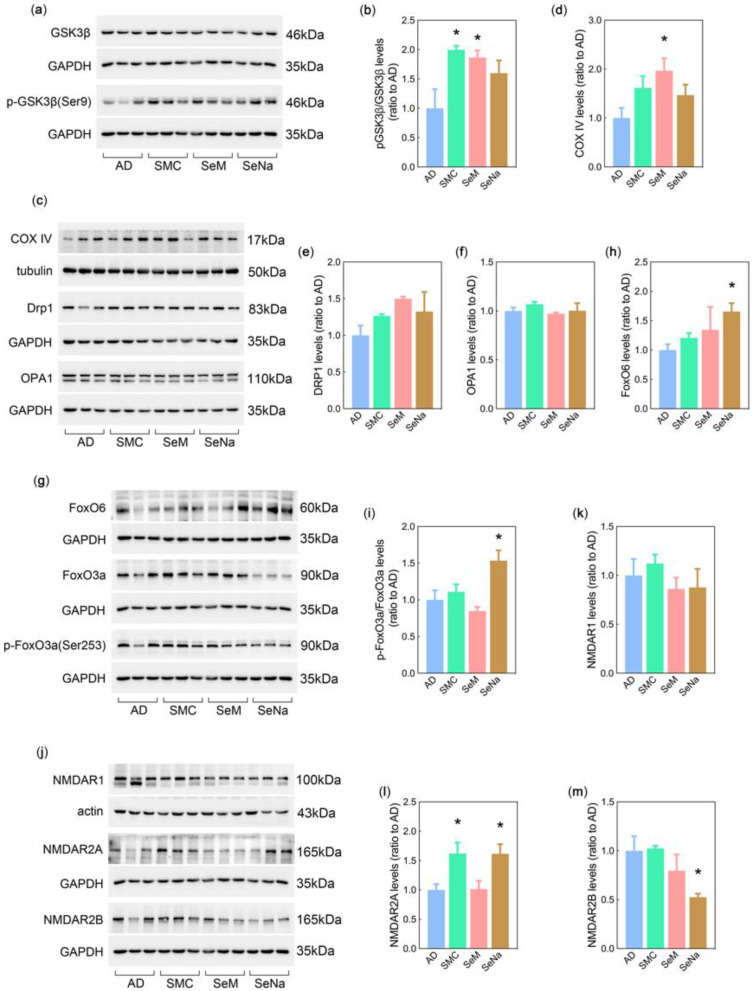
Levels of key proteins, kinases, and receptors in the signaling pathways related to AD pathologies. (**a**) Levels of GSK-3β and phosphorylated GSK-3β (Ser9) in the cortexes of 3× Tg-AD mouse brains were analyzed by Western blotting. (**b**) Quantification of protein levels in (**a**). (**c**) Levels of COX IV, Drp1, and OPA1 were analyzed by Western blotting. (**d**–**f**) Quantification of protein levels in (**c**). (**g**) Levels of FoxO6, FoxO3a, and phosphorylated FoxO3a (Ser253) were analyzed by Western blotting. (**h**,**i**) Quantification of protein levels in (**g**). (**j**) Levels of NMDAR1, NMDAR2A, and NMDAR2B were analyzed by Western blotting. (**k**–**m**) Quantification of protein levels in (**j**). α-Tubulin, β-actin, or GAPDH were used as loading controls. All data are presented as the means ± SEMs (*n* = 3). * *p* < 0.05 vs. AD mice, as determined by one-way ANOVA followed by Dunnett’s multiple comparison test.

To gain insight into the mechanisms of the three Se compounds related to their unique effects on AD pathology, kinases or proteins involved in regulating tau phosphorylation, mitochondrial quality, synaptogenesis, and glutamate receptors were explored, according to the results of the metabolomics analysis. The ratio of phosphorylated GSK-3β at Ser9 (the inhibitory site of its activity) to total GSK-3β was significantly higher in the SMC- and SeM-treated AD mice than in the untreated AD mice, whereas there was an upward trend in the SeNa group (Figure 5a,b). Although there were no significant changes in the levels of dynamin-related protein 1 (Drp1) and optic atrophy 1 (OPA1) in each group, SeM significantly increased the expression of cytochrome c oxidase complex IV (COX IV) in the 3× Tg-AD mice (Figure 5c–f). In addition, SeNa administration significantly increased Foxo6 and phosphorylated Foxo3a levels compared to those in the control group (Figure 5g,i). Excitatory glutamate receptors (N-methyl-D-aspartate receptor (NMDAR)) directly associated with synaptic plasticity were also detected, and the levels of NMDAR1 (constitutive subunit of NMDARs) were not affected by Se compounds (Figure 5j,k). For the functional subunits, NMDAR2A levels in the brains of AD mice treated with SMC and SeNa were significantly higher than those in control mice (Figure 5j,l). However, a significant reduction in NMDR2B levels was observed only in the SeNa group (Figure 5j,m). Therefore, these data suggest an attenuating effect of SMC and SeM on inhibition of GSK-3β activity, an ameliorating effect of SeM on mitochondrial function, and Foxo-signal-dependent SeNa effects on synaptic function, which further confirm the variable effects of the three Se compounds in improving AD pathology.

## 4. Discussion

Se is widely believed to be an essential micronutrient in the human brain, and its deficiency leads to cognitive decline [40]. There is increasing evidence that several Se compounds have therapeutic potential for AD. SMC is mainly found in seleno-containing plants, and studies have shown that SMC regulates oxidative stress and synaptic function in the brain, thereby improving cognitive impairment in AD mice [14,18]. SeM, the main chemical form of Se consumed by humans, alleviates cognitive impairment in AD mice by reducing Aβ deposition and tau hyperphosphorylation [17,21]. In addition, SeNa, as a representative inorganic Se compound, has been shown to improve AD pathology [41,42]. However, few studies have investigated the specific effects of different Se compounds on AD in vivo. Different Se compounds affect the expression and activity of different selenoenzymes and selenoproteins and may also have different effects on metabolic pathways in the body. More importantly, to ensure the therapeutic effect of Se in previous studies of AD, the Se was administered via long-term dietary supplementation at the upper limit of the safe dosage, which gave the illusion that almost all Se compounds were homogenous in terms of improving AD pathology. However, this is not the case, especially with the low-dose Se supplementation that is more acceptable for clinical use. Therefore, the purpose of this study was to explore the similarities and differences in the anti-AD effects of SMC, SeM, and SeNa at low doses and under short durations of administration to provide a reference for the potential clinical application of Se compounds in AD therapy.

Similar to observations in other studies, SMC, SeM, and SeNa alleviated cognitive impairment in 3× Tg-AD mice in the Morris water maze and Y-maze tests, even with low-dose Se supplementation for one month. In the open-field test, SMC and SeM significantly improved the autonomous behavior of AD mice, while the elevated plus maze test showed that SeM and SeNa could better ease the anxiety of AD mice. Importantly, we found differences in the improvement of major AD pathologies with low doses of the three Se compounds. SMC significantly reduced tau phosphorylation. The levels of Aβ oligomers were significantly decreased in the brains of AD mice treated with SeM, and a reduction in BACE1 expression was observed in the SMC- and SeM-treated groups. Additionally, SeNa significantly increased the levels of synaptic proteins (PSD95 and synaptophysin) in AD mice, and only synaptophysin expression was significantly elevated in the SeM group. These results suggest that different Se compounds exhibit preference and priority for AD pathology.

It is well-known that the main function of Se in the body, namely, its antioxidant effect, is directly correlated with Se levels, as well as several important selenoenzymes and selenoproteins, which might also be reflected in the ability of the three Se compounds to improve AD pathology. The data showed that SMC, SeM, and SeNa significantly increased Se levels in the brains of AD mice, although the effect of SeM was more significant. In addition, total GPx activity was enhanced with administration of each of the three Se compounds. However, assessment of the two major GPx enzymes (GPx4 and GPx1) in the brain showed that only SeM significantly increased GPX1 levels in AD mice, and no significant differences in the levels of GPx4 were observed among the experimental groups. TrxR activity and expression were significantly increased in AD mice treated with SMC. As for another selenoenzyme, MsrB1 (previously called SELENOR), its level was significantly increased in the SeNa-treated group. Interestingly, numerous studies have indicated an association between GPx1 and Aβ pathology [43,44], modulation of phosphorylated GSK-3β by TrxR activity [45,46], and the effects of SELENOR on synaptic function [47]. Therefore, our results indicate that different Se compounds induce the activity and expression of different selenoenzymes in the brains of AD mice, which may be the fundamental reason for their different effects on AD pathology.

Many studies have shown that disruption of redox homeostasis affects various biochemical metabolic pathways in the brain. In the metabolic profile analysis, we found that the pathological changes in some metabolites were significantly reversed in the brains of AD mice upon administration of the three Se compounds. The specific metabolites reversed by SMC, SeM, and SeNa were reported to be associated with the corresponding AD pathology in our study, indicating that changes in these metabolites were involved in the anti-AD process of each compound. Further analysis revealed that the three Se compounds reversed different metabolic pathways in the brains of AD mice. Among the metabolic pathways that SMC ameliorated, thiamine metabolism is believed to be involved in the phosphorylation of GSK-3β [30]. GSK-3β, an important glycogen synthase in the human body, is a major kinase involved in protein phosphorylation, and our results showed that SMC decreased tau phosphorylation by inhibiting the activity of GSK-3β. The glyoxylate and dicarboxylate metabolic pathways ameliorated by SeM are believed to be related to mitochondrial dysfunction, and their dysfunction leads to increased Aβ production in vivo, which is consistent with our results showing increased levels of COX IV (an enzyme complex of the mitochondrial respiratory chain) and reduced Aβ production in SeM-treated AD mice. The Foxo signaling pathway is closely related to memory consolidation and synaptic function, implying that restoration of synaptic deficits by SeNa might be related to this pathway. Subsequent quantification showed an enhancement of Foxo6 expression, as well as Foxo3 activity, as we speculated. 

In contrast, we found that AD mice treated with each of the three Se compounds showed a reversal in the metabolic pathways associated with amino acids. Amino acids are involved in the metabolism of various neurotransmitters and regulate inhibitory–excitatory homeostasis through synaptic receptors. The glutamate receptor, NMDAR, is one of the principal excitatory synaptic receptors responsible for the formation and maintenance of LTP. However, the imbalance between synaptic NMDAR (mainly NMDAR2A) and extra-synaptic NMDAR (mainly NMDAR2B) directly disturbs normal synaptic function, and this process is related to neurotransmitter amino acids (glycine and D-serine) [48]. Our results showed that SMC and SeNa promoted NMDAR2A expression, whereas SeNa and SeM decreased NMDAR2B levels. This may be a common mechanism by which Se compounds improve cognitive impairment in AD.

In summary, at low doses and under short durations of administration, SMC preferentially increased the expression and activity of thioredoxin reductase and reduced tau phosphorylation mediated by GSK-3β. SeM mainly affected GPx1 levels, thereby decreasing amyloid beta production and improving mitochondrial function. SeNa promoted the expression of MsrB1 and synaptic proteins to restore synaptic deficits. Our study provides evidence showing that different selenium compounds have different propensities for the treatment of AD pathology. Our study also elucidates the mechanisms and related targets that are responsible for these differences from the perspective of selenoenzymes and metabolic profiles.

## Figures and Tables

**Table 1:** Antibodies.

Reagent	Source	Identifier
Rabbit anti-BACE1	CST	5606
Mouse anti-Aβ	Biolegend	SIG-39300
Rabbit anti-APP	Abcam	ab32136
Mouse anti-Tau	Invitrogen	MN1000
Mouse anti-pSTau (Ser202)	Invitrogen	MN1020
Rabbit anti-pSTau (Ser202)	Abcam	Ab92676
Rabbit anti-PSD95	Abcam	ab76115
Rabbit anti-Synaptophysin	CST	4329s
Mouse anti-TrxR1	Santa Cruz	sc-28321
Rabbit anti-GPX4	Abcam	ab125066
Rabbit anti-GPX1	GeneTex	GTX116040
Rabbit anti-SELENOP	Abcam	ab109514
Rabbit anti-MSRB1 (SELENOR)	Invitrogen	Cat# PA5-77009
Mouse anti-GSK3β	CST	9832
Rabbit anti-pGSK3β (Ser9)	CST	9323
Rabbit anti-Foxo3a	CST	2497
Rabbit anti-pFoxo3a (Ser253)	CST	9466
Rabbit anti-Foxo6	Proteintech	19122
Rabbit anti-β-actin	Proteintech	81115
Mouse anti-α-tubulin	Proteintech	11224
Rabbit anti-GAPDH	Proteintech	10494
Goat anti-Rabbit IgG	CST	7074
Goat anti-Mouse IgG	CST	7076

## Data Availability

Data is contained within the article.

## Supplementary Materials

The following supporting information can be downloaded at: https://www.mdpi.com/article/10.3390/antiox12030702/s1, Figure S1: Score plots accurately distinguished WT, AD, SMC, SeM, and SeNa by OPLS-DA analysis; Figure S2: Clustering heatmap of the top 50 differential metabolites in the comparison groups; Figure S3: KEGG enrichment analysis of differential metabolites.
