# Supplementary figures and images for: Different Effects and Mechanisms of Selenium Compounds in Improving Pathology in Alzheimer’s Disease
Source: Antioxidants (Basel). 2023 Mar 12;12(3):702. doi: 10.3390/antiox12030702 (PMC10045564; doi:10.3390/antiox12030702)

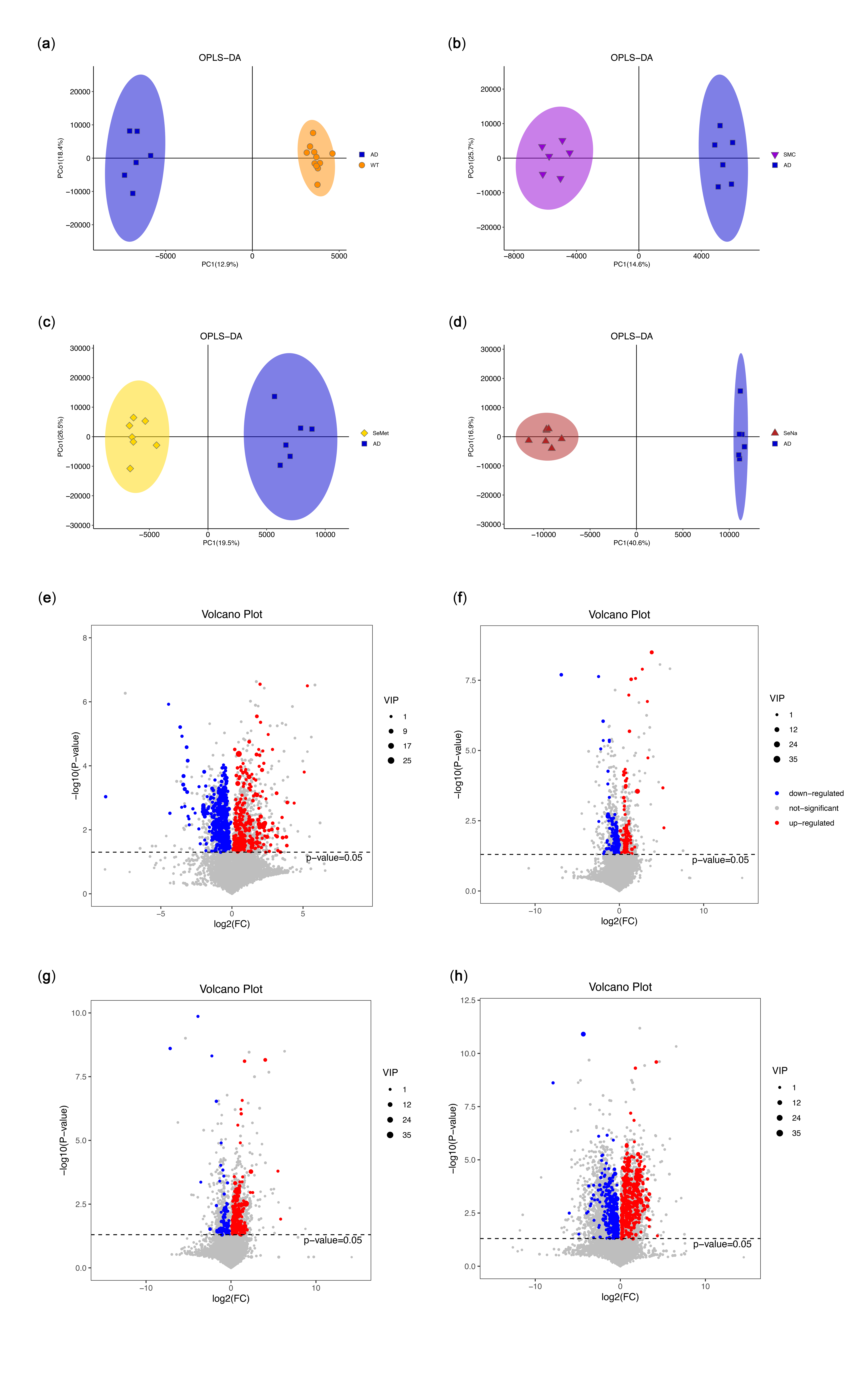

Supplement: Supplementary file 1 [file antioxidants-12-00702-s001.zip › Figure S1.tif]

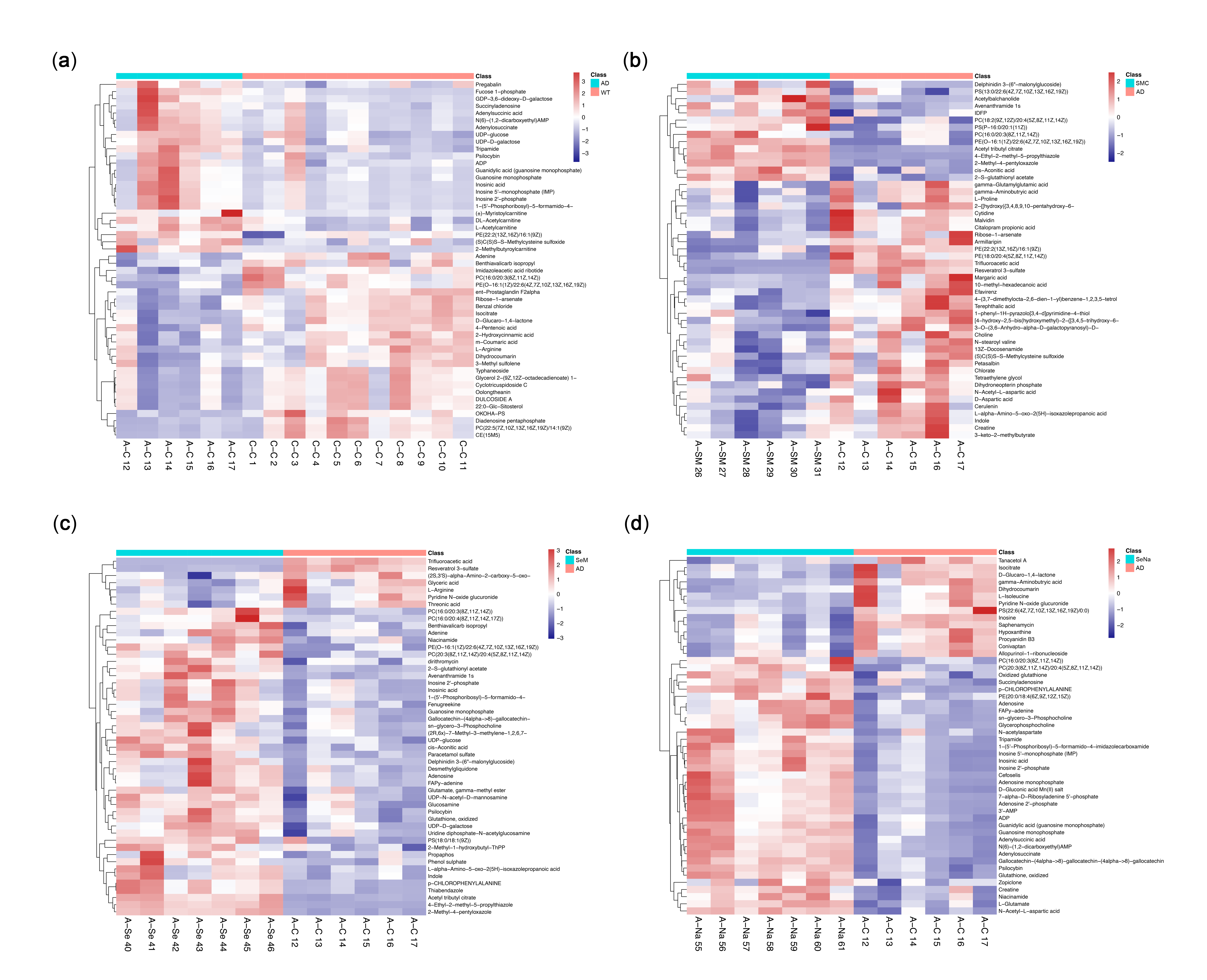

Supplement: Supplementary file 1 [file antioxidants-12-00702-s001.zip › Figure S2.tif]

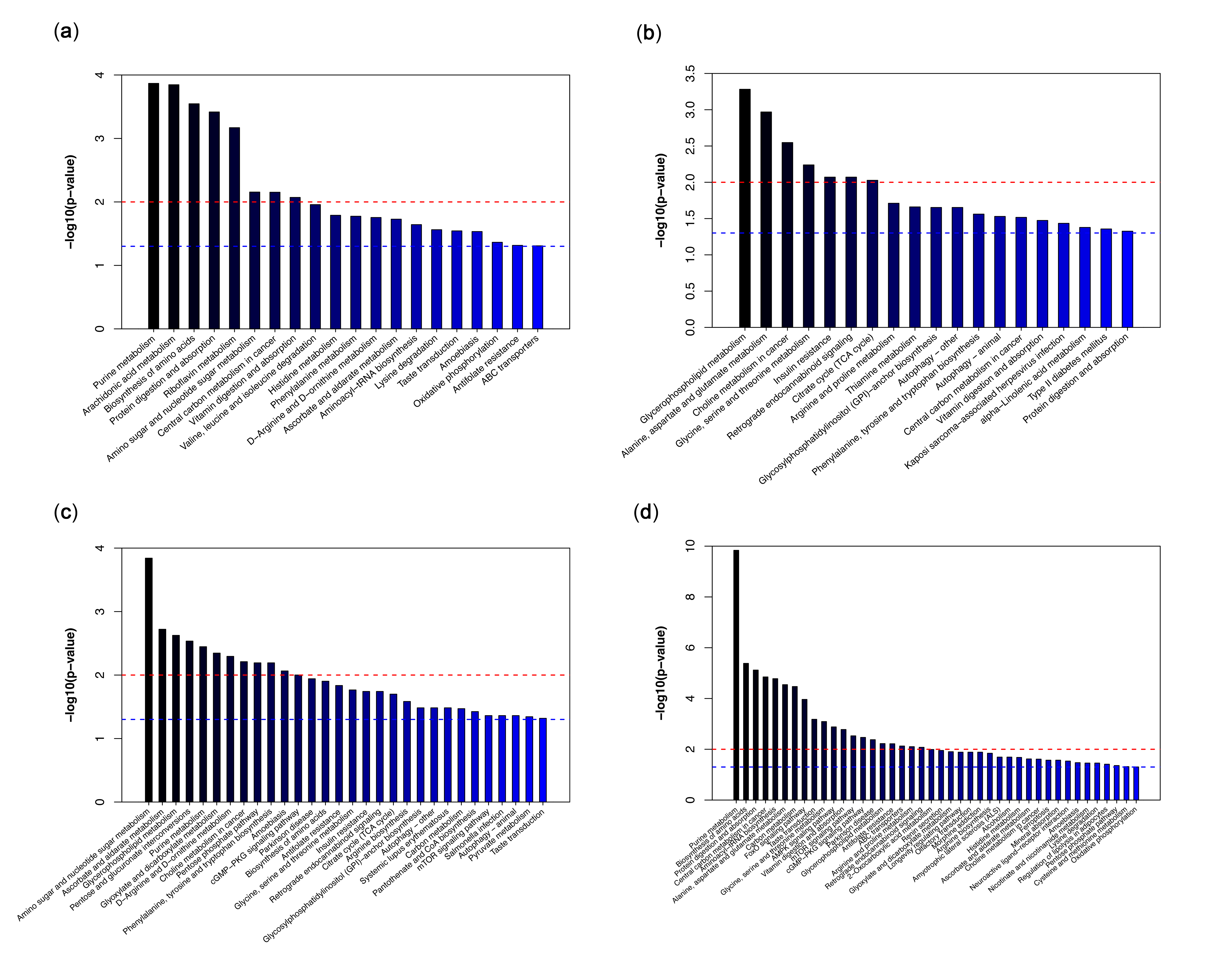

Supplement: Supplementary file 1 [file antioxidants-12-00702-s001.zip › Figure S3.tif]
